# Supplementary material for: The effects of genital myiasis on the diversity of the vaginal microbiota in female Bactrian camels
Source: BMC Vet Res. 2022 Mar 5;18:87. doi: 10.1186/s12917-022-03189-5 (PMC8897907; doi:10.1186/s12917-022-03189-5)
Supplement: Supplementary file 5 — Additional file 5. [file 12917_2022_3189_MOESM5_ESM.zip › MPL201709200_16s_yy/Treat1/C07_picrust/KEGG_L2_Organismal_Systems.pdf]

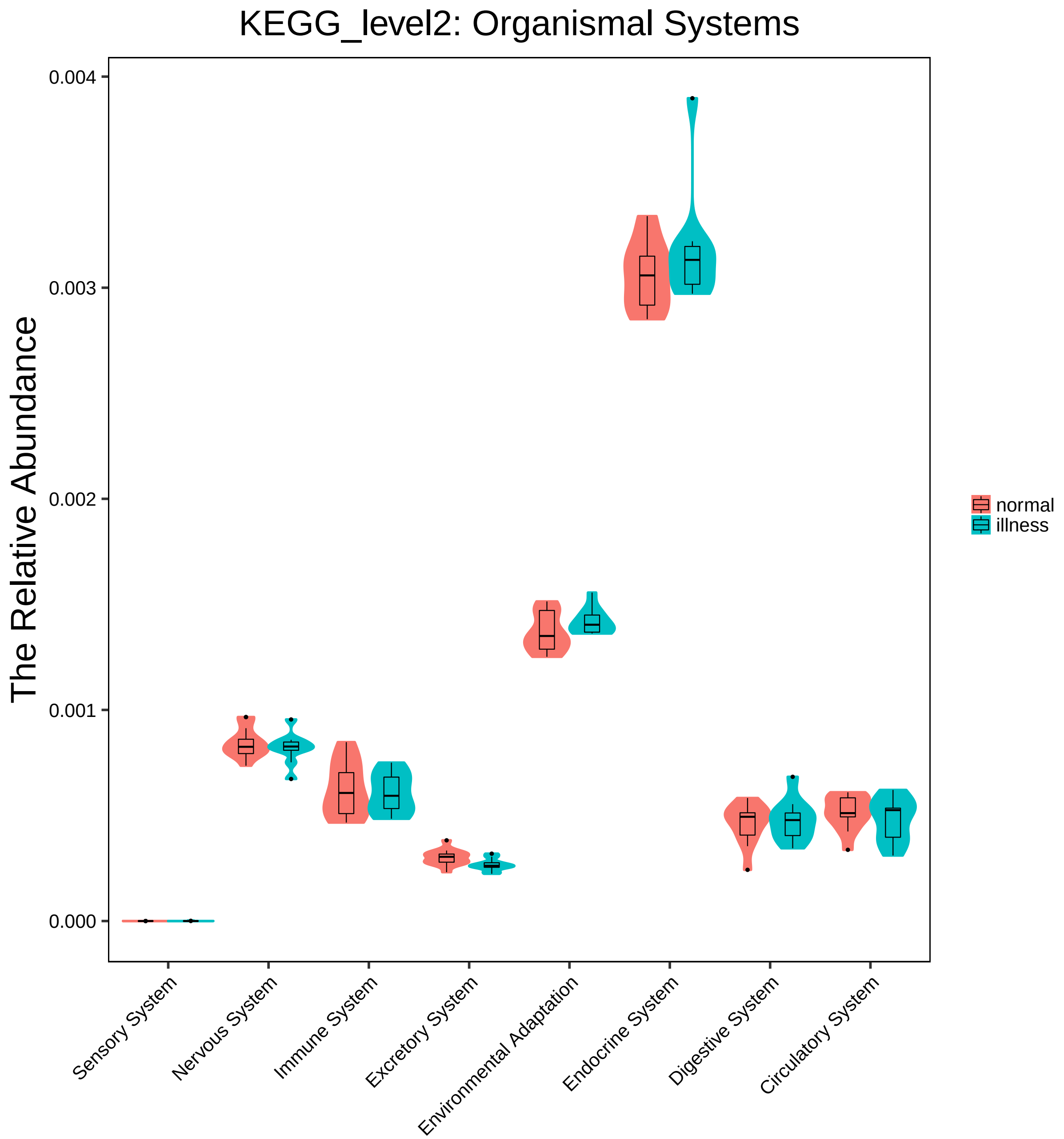

KEGG_level2: Organismal Systems
The Relative Abundance
0.004
0.003
0.002
0.001
0.000
Sensory System
Nervous System
Immune System
Excretory System
Environmental Adaptation
Endocrine System
Digestive System
Circulatory System
normal
illness
